# Fiberscope-Assisted Surfactant Therapy (FAST) in Neonatal Respiratory Distress Syndrome: Four-Year Retrospective Cohort Study

**DOI:** 10.3390/children13060755

**Published:** 2026-05-29

**Authors:** David Guevorkian, Eric Cavatorta, Yoann Marechal

**Affiliations:** 1 Neonatal Intensive Care Unit, HUMANI, CHU Marie Curie Public Hospital, Chaussée de Bruxelles 140, 6042 Charleroi, Belgium; eric.cavatorta@humani.be (E.C.); yoann.marechal@humani.be (Y.M.); 2 Medical Simulation Center (CACTUS), HUMANI, CHU Marie Curie Public Hospital, Chaussée de Bruxelles 140, 6042 Charleroi, Belgium

**Keywords:** FAST, surfactant replacement therapy, neonatal respiratory distress syndrome, endoscopy, INSURE

## Abstract

**Highlights:**

**What are the main findings?**

- We describe the clinical efficacy of a novel, minimally invasive surfactant therapy technique we developed, Fiberscope-Assisted Surfactant Therapy (FAST), in the management of neonatal respiratory distress syndrome.
- The comparison of FAST with INSURE (Intubation–Surfactant–Extubation) shows comparable respiratory efficacy and greater hemodynamic stability of FAST, with the advantage of being performed without sedation and analgesia.

**What are the implications of the main findings?**

- FAST is a promising and effective alternative for more comfortable surfactant therapy in preterm infants.
- Its minimally invasive nature would allow for early or prophylactic use and presents potential for integration into family-centered, skin-to-skin care practices.

**Abstract:**

**Background/Objective**: Surfactant replacement therapy remains a life-saving intervention in the management of neonatal respiratory distress syndrome (RDS). In 2022, we presented a novel minimally invasive method of surfactant delivery with a flexible endoscope: Fiberscope-Assisted Surfactant Therapy (FAST). This new study describes the clinical course of neonatal RDS in neonates treated with FAST, comparing them to those treated with INSURE (Intubation–Surfactant–Extubation). **Method**: In this single-center cohort study, we retrospectively analyzed data from neonates with RDS admitted to our unit between 2021 and 2024. Following surfactant therapy, short- and medium-term respiratory and hemodynamic effects were compared between the two cohorts. **Results**: Data from 21 neonates born at 27 to 35 weeks of gestation (birth weights 890–2685 g) treated with FAST and 37 neonates born at 27 to 35 weeks (birth weights 920–2910 g) treated with INSURE were analyzed. The two groups were comparable in gestational age and antenatal steroid exposure. In the FAST group, the procedure was successfully completed in all cases without sedation and analgesia, with a consistent trend toward reduced FiO_2_ and pCO_2_, as well as increased pH. Comparing FAST with INSURE, no significant differences were observed in respiratory outcomes, with a significant difference in the need for blood volume expansion after surfactant delivery (1 infant in FAST vs. 17 infants in INSURE; *p* < 0.001). **Conclusions**: FAST demonstrated comparable respiratory efficacy to INSURE, with greater hemodynamic stability without sedation and analgesia or laryngoscopy, making it an even less invasive option for surfactant therapy.

## 1. Introduction

Neonatal respiratory distress syndrome (RDS) is primarily caused by lung immaturity and endogenous surfactant deficiency in preterm infants. Since its introduction into neonatal care more than forty years ago [1], surfactant replacement therapy has remained a life-saving intervention in the management of RDS [2,3,4,5,6,7]. Based on current recommendations, if clinically indicated, administration of animal-derived surfactant (poractant alfa, 200 mg/kg) should not be delayed [8]. Over time, techniques for tracheal administration have evolved toward less invasive approaches. The INSURE (Intubation–Surfactant–Extubation) technique [9], used since the 1990s, and the LISA (Less Invasive Surfactant Administration) technique, introduced in the 2010s [10,11], are now widely implemented. Growing evidence suggests that LISA may be a preferable alternative to INSURE in spontaneously breathing preterm infants [12,13,14,15,16], offering at least similar efficacy [17,18,19,20,21,22,23]. Moreover, LISA avoids general sedation and analgesia, which is often required for INSURE, although both techniques involve laryngoscopy, known to be painful and potentially harmful. As a result, current protocols increasingly incorporate mild sedation during LISA to reduce the discomfort associated with laryngoscopy [24,25].

In a recent study, we presented a new technique for surfactant administration in spontaneously breathing neonates using a flexible endoscope instead of a laryngoscope: Fiberscope-Assisted Surfactant Therapy (FAST) [26]. This approach allows surfactant delivery without pharmacological sedation, minimizing procedural stress and maintaining spontaneous breathing. In that pilot study, we demonstrated its feasibility, safety, tolerability and short-term clinical benefits. We proposed FAST as a new form of minimally invasive surfactant therapy (MIST) for neonates with RDS, even less invasive than LISA or INSURE, as it requires neither laryngoscopy nor sedation and analgesia. Driven by these preliminary findings, we sought to further evaluate FAST in routine clinical practice. In this retrospective study, we compare the cohort of neonates who received surfactant via FAST since its regular adoption in our unit in 2021 with those treated by means of INSURE during the same period.

## 2. Materials and Methods

### 2.1. Study Design and Population

We conducted a single-center retrospective cohort study based on prospectively collected data from neonates admitted to the Level 3 Neonatal Intensive Care Unit (NICU) of CHU Marie Curie Public Hospital (Charleroi, Belgium) between 1 January 2021 and 31 December 2024. This study was conducted in accordance with the Declaration of Helsinki and Good Clinical Practice guidelines. The study protocol was approved by the Ethics Committee of CHU Marie Curie Public Hospital, Charleroi, Belgium (approval number P25/10_24/03, reference CCB B3252025000024). Given the retrospective nature of the study, and in line with the Ethics Committee’s decision, the requirement for informed consent was waived.

For many years, the standard method of surfactant administration in our NICU has been INSURE. Since the development of the FAST technique in 2021, some neonates have received surfactant using FAST, depending on the availability of neonatologists trained in the procedure at admission. Moderate and severe RDS were defined based on the FiO_2_ required to maintain SpO_2_ ≥ 90% with a PEEP of 5–6 cmH_2_O. Moderate RDS was defined as FiO_2_ 0.30–0.39, while severe RDS was defined as FiO_2_ ≥ 0.40. Poractant alfa (Curosurf^®^, Chiesi, Parma, Italy) at a dose of 200 mg/kg was administered as early as possible after diagnosis.

The first cohort consisted of neonates with RDS who received surfactant via FAST, following the procedure described in previous work [26]. In this new technique, applied to spontaneously breathing newborns without sedation and analgesia, surfactant is administered under visual control using a small, flexible endoscope, which guides a thin catheter through its operating channel. Throughout the FAST procedure, to maintain a continuous PEEP of 6 cmH_2_O and prevent hypoxemia, the infant received an air/oxygen flow of 10 L/min via a mask and a T-piece (Neo-Tee^®^, Mercury Medical, Clearwater, FL, USA). For this study, single-use flexible endoscopes (2.7 mm diameter, 1.2 mm operating channel, Ambu^®^ aScope™ 5 Broncho 2.7/1.2, Ballerup, Denmark) connected to a video monitor (Ambu^®^ aView™ 2 Advance) were used.

The infant was in the supine position. During FAST, patient comfort was ensured through non-pharmacological measures, such as optimized positioning and holding. The endoscope, containing a 2.5 French catheter with a 1.3 French tip (Perifix^®^ One, B. Braun, Melsungen, Germany) in its working channel, was inserted into the nostril through the mask via a connector (Bronch-Safe^®^ Swivel, Sontec Medical, Rochester, NH, USA). The endoscope was inserted until the vocal cords were visible, remaining above the larynx. Under visual control, the catheter was advanced by sliding it through the working channel to insert it into the trachea. Surfactant was then instilled slowly, while monitoring the infant’s tolerance. If surfactant reflux occurred, the delivery rate was adjusted, under visual control throughout the procedure. After surfactant administration, the endoscope with the catheter was removed for transition to nCPAP (https://doi.org/10.6084/m9.figshare.20653974.v1).

The second cohort comprised neonates born in the same gestational age range with RDS who received surfactant via INSURE. As premedication for intubation, neonates in the INSURE group received intravenous propofol (1–2 mg/kg, titrated). Infants were extubated as soon as effective spontaneous breathing was established, with a maximum duration of positive pressure ventilation of 30 min.

Medical records of eligible neonates were reviewed to identify surfactant administration cases and to extract relevant data on the procedure, short-term effects, and clinical course.

### 2.2. Data Collection and Analysis

To evaluate the short-term effects of the FAST and INSURE techniques, we recorded changes in FiO_2_, pCO_2_, and pH before and after surfactant administration, need for blood volume expansion after surfactant delivery, duration of respiratory support, duration of oxygen therapy, need for a second surfactant dose, and need for mechanical ventilation within the first 72 h of life.

No formal sample size calculation was performed for this study. Statistical analyses were performed using GraphPad Prism 10.5.0 (GraphPad Software, Boston, MA, USA). The normality of the collected data was assessed using the Shapiro–Wilk test. As most variables did not meet the assumption of normality, data are presented as median with interquartile range (IQR), and group comparisons were performed using the non-parametric Mann–Whitney U test. Categorial variables were summarized in contingency tables and compared between groups using Fisher’s exact test. A two-tailed *p*-value ≤ 0.05 was considered statistically significant.

## 3. Results

The FAST cohort included 21 neonates born between 27 and 35 weeks of gestation, with birth weights ranging from 890 to 2685 g. The INSURE cohort consisted of 37 neonates born between 27 and 35 weeks of gestation with birth weights ranging from 920 to 2910 g. The characteristics of the study population are presented in Table 1. The two cohorts were comparable in terms of gestational age, birth weight, antenatal corticosteroid exposure, cesarean delivery rate, FiO_2_ before surfactant therapy, and severe RDS rate. A higher proportion of male infants was observed in the FAST group; however, this difference reflects random variation, as intervention assignment was not selective.

In the FAST group, the procedure was successfully completed in all cases without any adverse events during FAST (such as injuries, technical difficulties, apneas, significant surfactant reflux, or the need for conversion to INSURE) without the need for sedation or analgesia. Following FAST, 20 of 21 infants showed a reduction in FiO_2_, 18 of 21 a reduction in pCO_2_, and 18 of 21 an increase in pH. Table 2 summarizes the respiratory and hemodynamic effects of surfactant therapy in both groups. Respiratory parameters before and after surfactant therapy are presented in the Table S1 in the Supplementary Material.

No statistically significant differences were found between the FAST and INSURE groups in terms of reduction in oxygen requirement, reduction in pCO_2_, increase in pH, duration of oxygen therapy, duration of respiratory support, CPAP failure requiring intubation within the first 72 h, and need for a second dose of surfactant. At the same time, the incidence of arterial hypotension requiring blood volume expansion was significantly lower in the FAST group (1 infant, 5%) compared with the INSURE group (17 infants, 46%); *p* < 0.001.

## 4. Discussion

Surfactant administration remains a cornerstone in the management of neonatal RDS. In our previous work, we described a new endoscopic technique developed in our NICU—Fiberscope-Assisted Surfactant Therapy (FAST)—which avoids the need for laryngoscopy and sedative drug administration, while offering additional advantages such as the maintenance of PEEP, visual guidance, control of surfactant reflux, and the use of small catheters (<1 mm). Following our pilot study in preterm infants > 28 weeks, FAST was adopted as a novel form of MIST and routinely implemented in our NICU from 2021 onwards. The availability of smaller diameter endoscopes now allows us to perform FAST even in premature babies less than 28 weeks (in the FATS group, there was 1 infant of 27 weeks and 3 infants of 28 weeks; the minimum weight was 1010 g). The present study retrospectively analyzed 21 neonates treated with FAST, comparing them to infants treated with INSURE during the same period, in order to confirm and expand upon our earlier findings.

Our results corroborate the initial pilot data, showing consistent respiratory improvement and excellent procedural safety with FAST. Importantly, there were no procedural failures, and, most notably, no sedation and analgesia was required. Given that it was a retrospective study, continuous recordings during the FAST procedure were not collected, so we cannot provide precise intra-procedural data. Although this retrospective analysis was not designed to demonstrate superiority or non-inferiority of FAST compared to INSURE, the observed outcomes were similar between the two groups. These findings suggest that FAST offers similar respiratory efficacy to INSURE, without formally stating the beneficial impact on overall neonatal morbidity, which was not analyzed for this study.

However, at this stage, it is difficult to generalize these findings since our investigation is limited to a single center and we do not have any newborns treated with LISA in our unit. The absence of statistically significant differences between groups may be influenced by the limited sample size, which could be considered as a limitation of this retrospective study. Further research would be valuable to compare FAST to INSURE and LISA on a larger scale. We are currently focusing on the design of such a study with other NICUs. This multicentric approach could better define FAST’s place among MIST techniques and potentially contribute to its wider implementation. It is worth noting that the current prices of single-use endoscopes remain a barrier to their routine use, hence our involvement in the development of cheaper equipment specifically for FAST with a bio-engineering laboratory.

Beyond efficacy, the main advantage of FAST is that it is even less invasive than INSURE or LISA, both of which require sedation and laryngoscopy, procedures that can be painful and potentially harmful. The development of FAST was driven by the desire to minimize these risks. Pain control and patient comfort during surfactant administration have always been priorities for neonatologists, given the potential for long-term negative effects of procedural stress. INSURE addresses discomfort through complete sedation, which ensures comfort but carries the disadvantage of exposure to anesthetic drugs. Challenges related to INSURE include the requirement for rapid laryngoscopy, intubation and surfactant administration in the absence of spontaneous breathing, brief positive pressure ventilation, potential failure of rapid extubation to CPAP, and the potential risk of hemodynamic instability as an adverse effect of sedation and analgesia.

A striking finding of this study was the markedly lower incidence of arterial hypotension requiring fluid expansion in the FAST group, likely reflecting the absence of anesthetic-induced hemodynamic collapse and instability. Thus, FAST avoids this threatening situation known for its risk of low cardiac output, hypoxia, metabolic acidosis and brain hemorrhage.

Interpretation of hemodynamic outcomes in neonatology must be cautious, given patient heterogeneity and variations in clinical practice. The difference observed in the need for blood volume expansion between the groups is probably related to the hemodynamic effects of propofol used for sedation in the INSURE group rather than to the technique itself or to an intrinsic patient factor. This should not be generalized to all sedative agents: each medication has its own potential adverse effects requiring specific corrective measures [15]. Reporting these outcomes remains clinically relevant, considering blood volume expansion as an indirect marker of the hemodynamic impact of sedation. We acknowledge that this observation is limited by the retrospective design, but it highlights a physiologically relevant signal that should be further investigated in a prospective interventional study, where sedation type can be standardized and its specific hemodynamic effects systematically assessed.

Since its introduction in the 2010s, LISA practice has evolved significantly [27]. Initially performed without sedation, an increasing number of NICUs now use mild sedation to improve tolerance [28]. The sedative agents are often the same as those used for INSURE (e.g., propofol, ketamine, fentanyl), given in lower doses to reduce the risks of apnea, respiratory depression, and CPAP failure. While studies on LISA with sedation have reported better comfort compared with no sedation, they also describe a proportion of potential adverse events including desaturation, surfactant reflux, apnea, the need for nasal positive pressure ventilation, and, in some cases, intubation and mechanical ventilation. Although the statistical and clinical relevance of these concerns still needs to be confirmed by future studies, their occurrence in daily practice warrants serious consideration and appropriate management [29,30]. Even if some of these events occur at similar rates with or without sedation, they may remain attributable to either respiratory depression caused by sedatives or to residual pain and vagal responses due to incomplete analgesia [31]. It is also important to note that medications routinely used for neonatal analgesia may carry both acute and long-term side effects [32].

Given that current LISA techniques still involve discomfort due to laryngoscopy, less invasive yet equally effective methods of surfactant administration could be preferable. Alternatives, such as nebulization, endoscopy, and the use of laryngeal mask airways [33,34,35,36] are under active investigation in both research and clinical settings. While encouraging data have been published, results remain insufficient to support their widespread implementation in routine practice. Surfactant nebulization, in particular, could offer a truly non-invasive method of surfactant delivery for preterm infants [37,38,39,40,41]. Growing evidence supports its potential role in the near future for the respiratory management of neonatal RDS. Achieving this goal will require optimization of nebulizer systems and nasal interfaces, careful selection of surfactant dose and type (synthetic or natural), and further clinical validation.

Until a fully non-invasive alternative becomes feasible, minimally invasive approaches like FAST remain highly relevant and aligned with current international recommendations for the management of neonatal RDS. Moreover, the minimally invasive nature of the FAST procedure, performed under spontaneous breathing, without laryngoscopy or sedation, opens up the possibility of integrating surfactant administration into a more physiological and family-centered care context. In particular, performing FAST while maintaining skin-to-skin contact between the parent and the preterm infant (‘kangaroo care”), as recommended by the WHO (World Health Organization) [42], could further enhance cardiorespiratory stability, thermal regulation, and parental bonding. This approach represents an exciting future direction for neonatal respiratory care, combining therapeutic efficacy with developmental and emotional benefits for infants and families.

## 5. Conclusions

This retrospective cohort study confirms our previous findings on the FAST technique, demonstrating consistent respiratory improvement following surfactant administration in preterm infants with RDS. Comparison with the INSURE group revealed similar respiratory outcomes, while FAST was associated with greater hemodynamic stability. Further large-scale prospective studies comparing FAST to LISA and INSURE are needed to validate these findings and explore this approach in broader clinical settings.

## Figures and Tables

**Table 1:** Study population characteristics.

	FAST (n = 21)	INSURE (n = 37)	*p*-Value (α = 0.05)
Gestational age (week), median (IQR)	32 (29–33)	31 (29–32)	0.34
Birth weight (g), mean (SD)	1760 (540)	1620 (478)	0.3
Gender (Male, %)	17 (81)	15 (41)	<0.01
Antenatal steroids (n, %)	14 (67)	23 (62)	0.78
Cesarean section (n, %)	15 (71)	18 (49)	0.76
FiO_2_ before surfactant, median (IQR)	0.3 (0.29–0.37)	0.31 (0.25–0.4)	0.70
Severe RDS (n, %)	5 (24)	11 (30)	0.86

IQR—interquartile range; SD—standard deviation; Severe RDS—FiO_2_ ≥ 0.40.

**Table 2:** Comparison of respiratory and hemodynamic parameters following FAST and INSURE.

	FAST (n = 21)	INSURE (n = 37)	*p*-Value (α = 0.05)
Δ O_2_ (%), median (IQR) ^1^	9 (6–17)	7 (4–12)	0.22
Δ pCO_2_ (mmHg), median (IQR) ^2^	8 (3–12)	4 (7–12)	0.21
Δ pH, median (IQR) ^3^	0.07 (0.03–0.1)	0.03 (−0.02–0.1)	0.11
O_2_ requirement (hours), median (IQR)	8 (4–35)	9 (6–48)	0.61
Respiratory support duration (days), median (IQR)	5 (2.5–9)	4 (2.5–26.5)	0.53
Invasive ventilation before 72 h (n, %)	1 (5)	3 (8)	0.99
Administration of second surfactant (n, %)	0	1 (2.7)	0.99
Blood volume expansion (n, %)	1 (5)	17 (46)	<0.001

^1^ Δ O_2_—calculated as difference in O_2_ % before and after surfactant therapy; ^2^ Δ pCO_2_—calculated as difference in pCO_2_ before and after surfactant therapy; ^3^ Δ pH—calculated as difference in pH after and before surfactant therapy; IQR—interquartile range.

## Data Availability

The data presented in this study are available from the corresponding author upon reasonable request due to legal reasons.

## Supplementary Materials

The following supporting information can be downloaded at: https://www.mdpi.com/article/10.3390/children13060755/s1, Table S1: Changes in respiratory parameters after surfactant therapy by FAST and INSURE.

## Abbreviations

The following abbreviations are used in this manuscript:

RDS	Neonatal respiratory distress syndrome
FAST	Fiberscope-Assisted Surfactant Therapy
INSURE	Intubation–Surfactant–Extubation
CPAP	Continuous positive airway pressure
FiO_2_	Fraction of inspired oxygen
PaCO_2_	Arterial partial pressure of CO_2_
PEEP	Positive end-expiratory pressure
SpO_2_	Peripheral oxygen saturation
LISA	Less Invasive Surfactant Administration
NICU	Neonatal intensive care unit
