# Supplementary material for: Fiberscope-Assisted Surfactant Therapy (FAST) in Neonatal Respiratory Distress Syndrome: Four-Year Retrospective Cohort Study
Source: Children (Basel). 2026 May 29;13(6):755. doi: 10.3390/children13060755 (PMC13296886; doi:10.3390/children13060755)
Supplement: Supplementary file 1 [file children-13-00755-s001.zip › children-4322510-supplementary.pdf]

Supplementary Table S1: Changes in respiratory parameters after surfactant therapy by FAST and INSURE.

	**FAST** ***(n=21)***	**INSURE** ***(n=37)***	**p-value** ***(α=0,05)***
**FiO_2 before surfactant**, *median (IQR)*	0,30 (0,29 – 0,37)	0,31 (0,25 – 0,40)	0,70
**FiO_2 after surfactant**, *median (IQR)*	0,21 (0,21 – 0,25)	0,23 (0,21 – 0,26)	0,15
**PCO_2 before surfactant** (mmHg), *median (IQR)*	60 (48 – 67)	58 (45 – 66)	0,77
**PCO_2 after surfactant** (mmHg), *median (IQR)*	50 (41 – 56)	52 (45 – 57)	0,46
**pH before surfactant**, *median (IQR)*	7.23 (7,17 – 7,26)	7,21 (7,17 – 7,30)	0,54
**pH after surfactant**, *median (IQR)*	7,29 (7,24 – 7,33)	7,27 (7,21 – 7,32)	0,21

IQR – interquartile range
